# Psychiatry

**Published:** 1923-03

**Authors:** J. O. Symes


### Progress of the Medical Sciences.

### PSYCHIATRY.

**Feeble-mindedness and Mental Deficiency (Amentia).**—Since the passing of the Mental Deficiency Act of 1913 increased attention has been paid to the diagnosis, classification, and treatment of the feeble-minded. Within the past few months there have appeared a fourth edition of Tredgold's book,[1] and a fifth edition of Shuttleworth and Potts' work.[2] Both these are standard text-books for the use of the medical profession, and deal with the subject more particularly from the scientific and medical standpoint; but the general public, and more particularly the teaching profession and psychologists, have been showing a keen interest in another side of the question, namely the value of intelligence tests in the detection of mentally deficient children, in the grading of backward children, and in the discovery of children of superior intelligence. There is no doubt that in this subject the educationist has outstripped the doctor, and this is a matter for regret. Binet and Simon's[3] little book is still one of the best guides for the busy practitioner, but it has been thought that the tests are better suited to French than to English children, and Terman's Stanford revision and extension of the Binet-Simon intelligence scale is now very generally adopted. A condensed form[4] of the bigger book was published in February, 1922, and should prove of great service. Additional tests are constantly being invented, and a full account and criticism of these and many new tests will be found in the weighty volume recently issued by the Education Office of the London County Council.[5]

The magnitude of the problem of the feeble-minded may be realised from the following facts: There are roughly 100,000 feeble-minded in England and Wales; of these over 30,000 are of school age, about one-half of whom are being educated in special schools at a cost of nearly half a million a year. The larger proportion of the feeble-minded population is dependent on the State from childhood. Few of the remainder are able to stand up to the competition of life, and between 20 and 30 years of age they too drift into institutions. Thirty per cent. of our workhouse population is feeble-minded, 10 per cent. of tramps, 10 per cent. of our asylum population, 25 per cent. of all women in workhouse maternity wards with illegitimate children, and ten per cent. of habitual criminals. Of the feeble-minded not in institutions it is found that in the country about one-half can earn a little, whilst in towns not more than a quarter can do so. One seldom or never finds a feeble-minded person earning a full wage. The burden on the State is one which will grow steadily and progressively, for the feeble-minded population grows more rapidly than the normal population, the average family of a feeble-minded woman being 7·5 as against the normal 4.3, while the mortality of such children up to 20 years of age is not greater than the average.

From a medical standpoint one of the most interesting chapters in Tredgold's book is that dealing with the pathology of amentia. He points out that the mental defect is the result of definite anatomical abnormalities, viz. numerical deficiency, irregular arrangement and imperfect development of the nerve cells of the brain, and that this arises from a defect of the germ cell which is inherited in the form of an innate predisposition to neuronic imperfection. Alcohol, tubercle and syphilis are the three chief agents in causing the germ degeneration. This view is one of fundamental importance, for it must profoundly influence both diagnosis and treatment. Only a small percentage of cases of feeble-mindedness are caused by grosser anatomical lesions, such as porencephaly, hydrocephaly, and mechanical and infective injuries to the brain at and shortly after birth.

The significance of this pathological finding is that it radically affects our views as to the aims and objects of treatment. It is obviously absurd to waste many years in trying to develop to a high standard the intellectual faculties of a person who is amented, and more and more attention will have to be paid to manual training. It was claimed for the Montessori[6] system that the idiot could be trained to the scholastic standard of the ordinary child, but even if this were correct it would be of little value if the child can never rise to an independent existence free from supervision and external support.

In the *diagnosis* of feeble-mindedness too much attention has been paid to the so-called " tests of intelligence." For the most part these are largely educational tests in disguise, and are best suited for the purpose of picking out children who are of exceptional mental ability or those who are backward. It cannot be too strongly emphasised that the diagnosis of amentia must be made on the lines of any other clinical diagnosis. First the family history must be carefully investigated, the personal history ascertained, with data as to the age at which dentition occurred, walking, and talking were begun, etc. Then a careful physical examination of the patient must be made in order that physical stigmata may be detected. Such stigmata are deformities of the head, jaws, teeth, fingers, or ears, evidence of poor nutrition, defective circulation, tics or habits. The last procedure is the application of mental efficiency tests, so as to determine the child's mental age. In our Bristol schools the intelligence tests as amended by Terman are in use, and the work is much simplified by the use of a printed sheet setting out the questions suitable for each age period, whilst spaces are provided for details, such as school proficiency, intelligence quotient, handwork, etc. Only by some such routine procedure is it possible to differentiate the feeble-minded from the imbecile or the idiot, and to classify the feeble-minded in groups according to their educational ability. The opinion must be formed on a survey of the whole facts of the case, not solely or chiefly on the result of mental efficiency tests. The difficulty in diagnosis is chiefly in distinguishing the high-class feeble-minded from the backward or retarded child. There is no hard and fast line between these two grades, just as there is no distinct line of demarcation between the idiot and the imbecile, or between the imbecile and the feeble-minded. In actual practice it is found that those children who are educated for a time in special schools, and are then capable of returning to the elementary schools, are nearly always backward and not mentally defective. In general terms the feeble-minded child cannot do tests higher than those set for 7 to 12 years of age. This is not always the case, however, and I have certainly known definitely mentally defective children who have reached such an educational standard as to enable them to keep their place in the elementary school, and indeed in some of the best public schools of the kingdom.

Much attention has been drawn lately to the subject of "moral imbecility" or "moral deficiency." Moral defectives are defined as "Persons who from an early age display some permanent mental defect, coupled with strong licentious or criminal propensities, on which punishment has had little or no deterrent effect." The greatest caution should be observed in making a diagnosis of moral deficiency, and the term should not be applied to the condition of the bulk of women who have illegitimate children in our Poor Law Infirmary wards, or to the many juvenile delinquents who appear over and over again in the police courts. Undoubtedly many of these are feeble-minded, but few are moral defectives. There is at the present time too great a tendency to attribute lapses of conduct to moral deficiency, rather than to the defects of training, environment, and self-control to which the feeble-minded are subject. It is, of course, most desirable that the proceedings of courts having jurisdiction over children should be regularly attended and watched by a medical officer skilled in the detection of mental deficiency. In this way many errors of justice may be avoided, and defective children who have escaped the vigilance of the Education Authorities may be brought under suitable control and supervision. It is much to be regretted that Bristol is behind other provincial cities in this matter. The work of Dr. A. W. Potts at the Birmingham courts is well known.

The *treatment* of the feeble-minded is two-fold in its purpose; firstly to secure the safety and happiness of the individual sufferer, and secondly to protect the community against a gradually-increasing population of feeble-minded who are not only a financial burden but, from their anti-social tendencies, may be a serious danger to the public welfare. It cannot be too strongly emphasised that the feeble-minded parent begets feeble-minded children. Tredgold quoted as an instance of this the well-known Kallikak family :—

" A certain Martin Kallikak married in 1837. Both he and his wife were normal, and their descendants for six generations, numbering several hundreds of individuals, were traced by Dr. Goddard, and were also normal. But Martin Kallikak had an illegitimate child by a girl who was feeble-minded, and the descendants along this line in about the same number of generations who were living under the same environment in the same State yielded no less than 222 feeble-minded offspring out of forty-one matings."

It follows from this that in the case of the greater number of feeble-minded of both sexes who have reached the age of puberty and who are not subject to close personal supervision, one of two courses must be taken. Either they must be sterilised or they must be segregated. Popular sentiment in this country is opposed to the former course, and public opinion must be educated further in the matter. In America sterilisation of defectives has been practised since 1907. Furber states that the sterilisation of mental defectives has been practised in certain of the United States since 1907. Twelve States have enacted such laws, but in New York and Michigan States they have been repealed. Oregon State refuses to issue marriage licences unless the defectives submit to sterilisation. The total number of operations since 1907 is 4,313. The operations selected were, in the male, division of the vas deferens, and in the female ligature of the Fallopian tubes. There is need at the present time of legal enactment forbidding the marriage of any feeble-minded person.

It has been suggested that many high-grade feeble-minded adults [7] might reside in hostels from which they might go to their daily work. The head of the hostel would advise them in monetary affairs and concerning employment, and would exercise a needed control over their behaviour. At the present time there are various farm colonies, laundry homes, and industrial institutions for the reception of adults who are certified under the Mental Deficiency Act, but the accommodation is totally inadequate, and to a very large extent defective children after reaching the age of sixteen years are drafted back to the imbecile wards of our Poor Law Institutions and County Asylums. The problem of the way in which the adult feeble-minded can be most profitably employed and segregated has yet to be solved.

The conditions in the case of juveniles are much better, and constantly improving. Everyone agrees that where home influences are favourable the defective child derives most benefit, and makes most progress, under the love and care of its own parents, and for these the special day schools are available. There is need, however, of training centres at which non-certified feeble-minded children beyond school age could be trained in domestic duties, or taught simple handicrafts, whilst still remaining under parental supervision.

A few of the institutions for the reception of defective children are philanthropic ventures, but for the most part they are provided by Boards of Guardians, at the request of the borough councils, and are supervised by the Board of Control. This division of responsibility does not make for efficiency or economy of working. The principle adopted is that of the education of the senses—dancing, singing, music, modelling, and appreciation of shade, weight and colour. Later manual occupations are introduced—gardening, farm work, boot and mat making, cooking, laundry work and mending. By these methods the child's intelligence is gradually awakened and then developed to its maximum value. The great difficulty at the present time is to find teachers who are trained to do this kind of work, and who are willing to undertake the tedious and difficult task of educating the defective child.

Operative treatment for microcephalic and hydrocephalic cases has now been abandoned. Organotherapy is practically confined to the use of thyroid in cretinoid cases. Psychotherapy has been tried in emotional cases, psychic epilepsy, and to control dirty habits, but has met with little success.

J. O. SYMES.

REFERENCES.

[1] Tredgold, A. F., *Mental Deficiency* (*Amentia*), 4th Edition; Baillière, Tindall & Cox, London.

[2] Shuttleworth, G. E., and Potts, W. A., *Mentally Deficient Children: their Treatment and Training*, 5th Edition, 1922.

[3] Binet and Simon, *Mentally Defective Children*, 1914; Edward Arnold, London.

[4] Terman, Lewis M., *The Measurement of Intelligence;* George G. Harrap & Co. Ltd.

[5] Burt, Cyril, *Mental and Scholastic Tests*, Report by Education Officer of the London County Council; F. S. King & Son Ltd.

[6] Culverwell, E. P., *The Montessori Principles and Practice;* G. Bell & Sons Ltd.

[7] Report of Conference on Mental Deficiency. *Brit. M. J.*, 1922, ii. 222.

---

